# Quality Cost A* Path Planning for Multi-Sensor Fusion in Corridor Smoke Scenarios

**DOI:** 10.3390/s26144530

**Published:** 2026-07-17

**Authors:** Yang Feng, Shuai Zhu, Letian Liu, Xin Liu, Hua Xia, Bingkun Zhang, Hao Chen, Ben Wang, Yan Sun

**Affiliations:** 1 School of Information Science and Engineering (SISE), Hangzhou Normal University, Hangzhou 311121, China; fengyang@hznu.edu.cn (Y.F.); zhushuai@stu.hznu.edu.cn (S.Z.); liuletian@stu.hznu.edu.cn (L.L.); xiahua@stu.hznu.edu.cn (H.X.); zhangbingkun08@stu.hznu.edu.cn (B.Z.); chenhao@stu.hznu.edu.cn (H.C.); 2 Mobile Health Management System Engineering Research Center of the Ministry of Education, Hangzhou 311121, China; 3 Zhejiang-Cyprus Smart City and Mobile Health Joint Laboratory, Hangzhou 311121, China; 4 The Center for Caribbean Studies, Hangzhou Normal University, Hangzhou 311121, China; 5 Hangzhou Juqi Information Technology Co., Ltd., Hangzhou 311121, China; liuxin@hzjqit.com; 6 Zhejiang Evaluation Center for Medical Service and Administration, Hangzhou 311121, China

**Keywords:** smoke scenarios, multi-sensor fusion, Beer-Lambert law, FDS, improved A* algorithm

## Abstract

Indoor fire smoke degrades visible-light cameras and near-infrared Lidar through wavelength-dependent absorption and scattering, threatening robotic navigation safety. Existing path planners either ignore sensor degradation or rely on empirical penalties lacking a physical basis. To address these issues, this paper proposes Quality Cost A* (QC-A*), which maps Fire Dynamics Simulator (FDS) visibility fields to sensor perception quality via the Koschmieder and Beer–Lambert physical laws, embedding a cost function that drives paths away from high-attenuation regions. A multi-sensor fusion layer provides fault tolerance under sensor-specific failure conditions. The method is validated through FDS-based simulations across four smoke scenarios in a 20 m × 6 m corridor with 21 obstacles, using 50 start–goal pairs per scenario. Perception quality derives from Beer–Lambert optical transmittance, while the hazard-zone proportion quantifies path segments with visibility below 5 m. Across the Symmetric and Asymmetric scenarios, QC-A* reduces the low-visibility hazard-zone proportion from 40.7% to 19.6% and improves worst-case perception quality from 0.067 to 0.177, with a 15.3% path length increase, while remaining close to traditional A* in light-smoke conditions. Under constructed sensor failure tests, QC-A* maintains a 96–100% planning success rate versus 48% for Camera-Only and 70% for Lidar-Only. QC-A* shifts sensor degradation modeling from empirical penalty to physical mechanism, achieving a favorable safety–efficiency balance prioritizing perceptual safety, and provides an interpretable, generalizable framework for robotic fire-environment path planning.

## 1. Introduction

Indoor fire smoke poses a serious challenge to mobile robotic navigation because it simultaneously changes the traversable environment and degrades onboard perception sensors. Smoke contains soot aerosols that attenuate electromagnetic radiation through wavelength-dependent absorption and scattering, reducing the effective visibility of optical sensing systems [1,2,3]. For visible-light cameras, smoke can cause image contrast reduction, feature loss, unstable feature matching, and failure of visual detection or localization. Near-infrared LiDAR is generally less sensitive to visible-band attenuation, but dense smoke particles can still weaken, scatter, or contaminate returned signals, thereby affecting obstacle detection and mapping reliability [3,4]. These effects are particularly critical in indoor corridor environments, where space is constrained, alternative routes are limited, and local perception errors may directly affect navigation safety. Therefore, for robots operating in smoke-filled indoor environments, path planning should not only consider geometric feasibility but also the spatial distribution of sensor perception quality.

Previous fire-related navigation and evacuation studies have shown that the safest path is not necessarily the shortest path. In building and tunnel environments, evacuation performance is affected by building layout, pedestrian movement rules, smoke development, fire risk, and route availability [5,6,7]. Cellular-automata-based and room-scale evacuation models have been used to represent indoor movement in discretized spaces and to evaluate route safety under constrained layouts [6,8]. Recent studies on intelligent firefighting and indoor evacuation route determination further indicate that route planning in fire environments should account for dynamic risk and changing environmental conditions rather than relying only on static distance metrics [9,10]. These studies provide an important basis for risk-aware planning in fire scenarios. However, most of them focus on human evacuation or route selection, whereas mobile robots must additionally consider whether their onboard sensors remain reliable along the planned route. In other words, for robotic navigation, environmental risk is not only related to exposure to smoke or fire, but also to whether the robot can maintain sufficient perception capability to execute the planned path safely.

Robot path planning has been extensively studied using graph-search, sampling-based, optimization-based, and hybrid planning frameworks. The classical A* algorithm provides an efficient and interpretable heuristic search structure for grid-based path planning [11]. Heuristic robot path-planning methods are widely used because they offer a balance between computational efficiency and path quality [12]. Sampling-based optimal planners, such as PRM* and RRT*, have also provided important theoretical foundations for motion planning in continuous and high-dimensional configuration spaces [13]. In recent years, improved A*-based methods have enhanced search efficiency and path quality through adaptive cost functions, bidirectional search, path smoothing, and global–local fusion strategies [14,15,16]. Other representative methods, including A*-DWA fusion, improved RRT-DWA frameworks, and A*-artificial potential field combinations, have also been developed to improve obstacle avoidance, path smoothness, and planning robustness [17,18,19]. Nevertheless, these methods mainly optimize geometric distance, obstacle clearance, turning smoothness, or computational efficiency. The influence of smoke-induced sensor degradation on path executability is still insufficiently considered. As a result, a geometrically feasible path may still be unsafe if it passes through regions where the robot’s camera or LiDAR perception is severely degraded.

The need to consider perception degradation is especially important in fire and smoke environments. Previous experimental studies have evaluated the performance of navigation sensors in fire smoke and confirmed that smoke can significantly affect robotic sensing reliability [4]. Recent robotic perception studies in degraded environments have also explored thermal SLAM, smoke-adaptive odometry, and thermal-inertial SLAM to improve localization and mapping under poor visibility or smoky conditions [20,21,22]. These studies demonstrate that perception robustness is a key requirement for robots operating in visually degraded environments. However, most existing works focus on improving perception or localization modules after sensor degradation has occurred. Relatively less attention has been paid to embedding physically modeled sensor perception quality directly into the path-planning cost function. From a planning perspective, it is preferable to avoid highly degraded regions in advance when alternative paths are available, rather than relying only on downstream perception algorithms to compensate for severe sensor degradation.

This study focuses on global path planning in known or pre-mapped indoor corridor environments, where the occupancy map is available and the smoke visibility field is treated as a planning-time input obtained from Fire Dynamics Simulator (FDS) simulation or updated perception. The proposed method is not intended to replace SLAM, local obstacle avoidance, or real-time perception modules. Instead, it aims to provide a physically interpretable global path planner that avoids regions where camera and LiDAR perception are expected to deteriorate severely. To this end, FDS visibility is first converted into smoke concentration using the Koschmieder visibility law and then mapped to sensor-specific optical quality through the Beer–Lambert extinction law [1,23,24]. The camera and near-infrared LiDAR quality fields are then fused into a multi-sensor perception-quality field, which is embedded into the A* step-cost function. This modeling chain establishes a connection between fire-smoke simulation, optical sensor degradation, multi-sensor fusion, and path-planning decision making.

Based on this idea, this paper proposes a Quality Cost A* path-planning method, termed QC-A*, for multi-sensor fusion in corridor smoke scenarios. Unlike conventional A* methods that primarily search for geometrically short paths, QC-A* introduces a physically derived perception-quality penalty into the path cost. As a result, the planner can balance path length, obstacle clearance, turning smoothness, and smoke-induced perception reliability. This design retains the interpretability and computational structure of classical heuristic search while enabling perception-aware planning in degraded indoor fire environments. The proposed method therefore provides a practical bridge between traditional grid-based path planning and sensor-quality-aware navigation in smoke-filled environments.

The main contributions of this paper are summarized as follows:

(1) A physical derivation chain from FDS visibility to sensor perception quality is established, converting FDS-generated visibility into smoke concentration and then into sensor-specific optical quality using the Koschmieder and Beer–Lambert laws [1,23,24];
(2) The sensor-specific quality fields are fused into the fused perception quality and incorporated into the QC-A* step cost, enabling perception-aware path planning based on physically modeled sensor degradation;
(3) Across the Symmetric and Asymmetric scenarios, QC-A* reduces the low-visibility hazard-zone proportion from 40.7% to 19.6% while increasing path length by 15.3%, and remains close to traditional A* with negligible additional path length in the Light-Smoke scenario;
(4) Under constructed sensor-specific failure conditions, QC-A* maintains a planning success rate of 96–100%, whereas Camera-Only decreases to 48% in the Symmetric scenario and Lidar-Only decreases to 70% in Fast-Spread, demonstrating the robustness provided by multi-sensor fusion.

The remainder of this paper is organized as follows. Section 2 describes the simulation environment and the physical modeling of sensor degradation. Section 3 presents the QC-A* cost function and algorithm flow. Section 4 reports the experimental validation, including parameter sensitivity analysis, path visualization, statistical comparison, generalization testing, and constructed sensor-failure stress tests. Section 5 concludes the paper and discusses future work.

## 2. Environment Construction and Sensor Degradation Physical Modeling

### 2.1. Simulation Environment Construction

**Figure 1:**
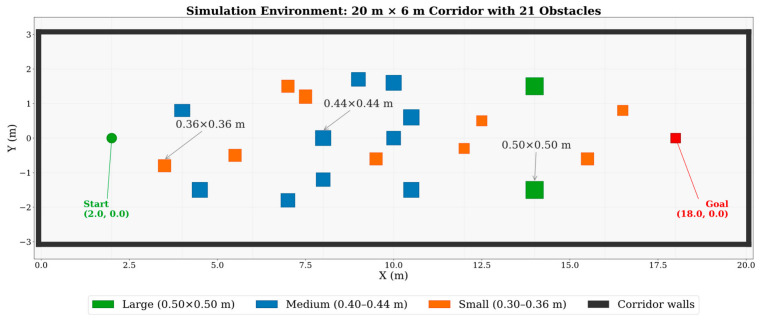
Simulation environment setup: 20 m × 6 m corridor with 21 obstacles. The green dot marks the start, and the red square marks the goal.

The simulation scenario is a 20 m×6 m corridor with a grid resolution of 0.1 m (200 × 60 cells), and both ends are open. Twenty-one obstacles are placed within the corridor (as shown in Figure 1). Each cell stores occupancy status, FDS visibility V(x,y), and perception quality Q(x,y), collectively forming the state space for A* search.

### 2.2. Physical Modeling of Sensor Perception Quality

Optical sensor degradation in smoke is modeled using the Koschmieder visibility law [1] and the Beer–Lambert extinction law [23]. NIST FDS 6.9.1 [24] is employed to simulate combustion-driven smoke transport and provide the spatial visibility field V(x,y) at the sensor height. Under the Koschmieder relationship, visibility is related to the smoke mass concentration by

(1) $$V(x,y)=\frac{K}{\sigma_{vis}C(x,y)}$$

where K=3.0 is the visibility constant adopted by FDS for light-reflecting objects under the Koschmieder visibility formulation [1,24], $\sigma_{vis}$ is the visible-band soot mass extinction coefficient, and C(x,y) is the local smoke mass concentration. The value $\sigma_{vis}$ = 8.7 $m^{2}/g$, measured at the representative visible wavelength of approximately 633 nm, is adopted following previous experimental studies and the FDS convention [2,24].

Rearranging Equation (1), the smoke mass concentration field is obtained from the FDS visibility output as

(2) $$C(x,y)=\frac{K}{\sigma_{vis}V(x,y)}$$

The reference distance $d_{ref}$ = 3.5 m is selected as a representative forward perception distance for corridor navigation rather than as the maximum sensing range of either sensor. The same value is used for both sensors to provide a unified basis for comparing wavelength-dependent attenuation. For the visible-light camera model, perception quality is defined as the optical transmittance over this distance:

(3) $$\begin{array}{ll} Q_{cam}(x,y) & =exp\left[-\sigma_{vis}C(x,y)d_{ref}\right]\\ \; & =exp\left[-\frac{Kd_{ref}}{V(x,y)}\right] \end{array}$$

here, $Q_{cam}\in (0,1]$, where a value close to 1 indicates weak attenuation and a value close to 0 indicates severe visible-light degradation.

For the near-infrared Lidar model operating at 905 nm, the soot mass extinction coefficient is estimated from the visible-band coefficient using inverse wavelength scaling:

(4) $$\begin{array}{cc} \sigma_{nir} & =\sigma_{vis}\frac{\lambda_{vis}}{\lambda_{nir}}\\ \; & =8.7\times \frac{633}{905}\approx 6.1\;m^{2}/g \end{array}$$

This approximation reflects the experimentally observed decrease in soot extinction toward the near-infrared spectral range [3]. The Lidar perception quality is therefore defined as

(5) $$\begin{array}{ll} Q_{lidar}(x,y) & =exp\left[-\sigma_{nir}C(x,y)d_{ref}\right]\\ \; & =exp\left[-\frac{\sigma_{nir}}{\sigma_{vis}}\frac{Kd_{ref}}{V(x,y)}\right] \end{array}$$

Combining Equations (3) and (5) gives

(6) $$Q_{lidar}(x,y)=Q_{cam}(x,y{)}^{\sigma_{nir}/\sigma_{vis}}\approx Q_{cam}(x,y{)}^{0.70}$$

Thus, under pure-smoke attenuation, the camera perception quality $Q_{cam}$ and the Lidar perception quality $Q_{lidar}$ have identical spatial ordering, although the Lidar quality decreases more slowly because of its lower adopted extinction coefficient. To prevent numerical underflow and excessively large downstream penalties, both quality values are clipped to the interval [0.001,1.0].

The quantities $Q_{cam}$ and $Q_{lidar}$ represent physical-layer optical quality rather than fully calibrated sensor-success probabilities. Actual sensor performance is additionally affected by detector noise, dynamic range, signal-processing algorithms, target reflectivity, and other hardware-dependent factors. To obtain a unified indicator for path planning, an availability-inspired fusion score is defined as

(7) $$Q_{fused}(x,y)=1-\left[1-Q_{cam}(x,y)\right]\left[1-Q_{lidar}(x,y)\right]$$

Equation (7) provides a monotonic surrogate for the availability of at least one sensing modality. It is not interpreted as a strictly calibrated probability under pure-smoke conditions because the two quality fields are correlated through the same visibility field. Its complementary value becomes most relevant when sensor-specific failure mechanisms, such as camera glare or Lidar degradation caused by water mist, disrupt this correlation.

The resulting physical modeling chain can be summarized as

(8) $$V(x,y)⟶C(x,y)⟶\left\{Q_{cam}(x,y),Q_{lidar}(x,y)\right\}⟶Q_{fused}(x,y)$$

**Figure 2:**
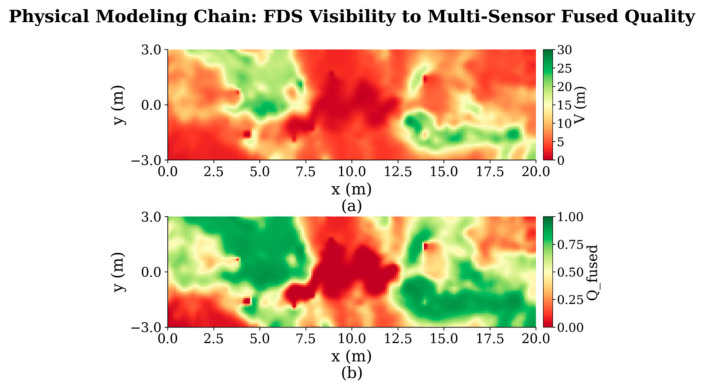
Physical modeling chain—from FDS visibility field to multi-sensor fusion quality field. (**a**) FDS visibility V(x,y); (**b**) multi-sensor fused perception quality $Q_{fused}(x,y)$.

The principal parameters used in this modeling chain are summarized in Table 1, while Figure 2 compares the spatial distributions of the original FDS visibility field and the resulting fused quality field.

## 3. QC-A* Algorithm Design

Having completed the physical modeling of sensor perception quality, this section describes how $Q_{fused}$ is embedded into the A* search framework so that path planning simultaneously considers geometric distance and sensor perception quality.

### 3.1. Quality Cost Function Design

Traditional A* [11] guides node expansion using the evaluation function f(n)=g(n)+h(n), where g(n) is the cumulative cost from the start node and h(n) is the heuristic estimate of the target. In the proposed cost function, c denotes the current node, n denotes a candidate neighboring node, and d(c,n) is the traversal distance from c to n. This paper embeds $Q_{fused}$ into the step cost and redefines g(n) as

(9) $$\begin{array}{ll} g(n)=g(c) & +d(c,n)\left[1+\alpha \left(1-Q_{fused}(n)\right)\right]\\ \; & +\beta_{blind}\max\left(0,\frac{Q_{blind}-Q_{fused}(n)}{Q_{blind}}\right)\\ \; & +\beta_{turn}\theta d(c,n)+s(n). \end{array}$$

The quality penalty term $d\left(c,n\right)\cdot [1+\alpha (1-Q_{fused}(n))]$ is the multiplicative core of the cost function. The default value α=3.0 is selected as a moderate trade-off coefficient between path length and perception quality and is further examined in the sensitivity analysis in Section 4.2. When $Q_{fused}$ approaches 1, the cost approaches the geometric distance d(c,n); when $Q_{fused}$ approaches 0, it increases to 4d(c,n). The multiplicative formulation reflects the assumption that a longer traversal through a degraded region produces greater cumulative perception risk.

$Q_{blind}$ is the perception blind-zone threshold, and θ is the heading-change angle between the previous movement direction and the current candidate step. The blind-zone penalty is activated when $Q_{fused}<0.25$, with $\beta_{blind}=50.0$. For example, the penalty is 2.0 at $Q_{fused}=0.24$ and 48.0 at $Q_{fused}=0.01$. Its magnitude substantially exceeds the geometric cost of a single grid step, thereby strongly discouraging the planner from entering perception blind zones. The gradient form avoids an abrupt cost change at $Q_{blind}$ while retaining limited passability under extreme conditions in which no alternative route exists.

The turn-smoothing term $\beta_{turn}\cdot \theta \cdot d(c,n)$, where $\beta_{turn}=0.02$, penalizes changes in movement direction and suppresses zigzag paths. The safety-distance term s(n) is computed using the safety radius $d_{safe}=0.3\;m$, which defines the neighborhood around obstacles where clearance penalties are applied. These two terms are integrated into the same cost function to improve the geometric quality and executability of the generated path. The remaining parameters are held constant across all experiments. These values respectively control blind-zone avoidance, path smoothness, obstacle clearance, the perception-failure threshold, and cumulative exposure. No scenario-specific parameter adjustment is performed.

### 3.2. Search Behavior and Adaptive Characteristics

Depending on the numerical distribution of $Q_{fused}$, Equation (9) produces three characteristic search behaviors. When $Q_{fused}>0.65$, the blind-zone penalty remains inactive, and the quality penalty is relatively limited. The resulting QC-A* path therefore tends to remain close to the traditional A* path without substantial additional detours. When $Q_{fused}$ lies between 0.25 and 0.65, the quality penalty progressively increases as $Q_{fused}$ decreases. The planner balances path length against perception quality and may take moderate detours to avoid more severely degraded regions. When $Q_{fused}<0.25$, the blind-zone penalty dominates the step cost. The planner therefore strongly avoids such regions whenever an alternative route is available and traverses them only under extreme conditions in which no feasible alternative exists.

These three intervals describe the search behavior induced continuously by Equation (9), rather than representing three independently switched planning modes. Among the cost parameters, α is the primary parameter controlling the trade-off between path length and perception quality. The remaining thresholds are fixed according to task-safety requirements, robot clearance, and exposure constraints, and the same values are used across all experiments without scenario-specific tuning.

### 3.3. Multi-Sensor Fusion Embedding Strategy

The motivation for using $Q_{fused}$, rather than a single-sensor quality value, is to exploit the complementary degradation characteristics of visible-light cameras and near-infrared Lidar. The availability-inspired fusion function in Equation (7) produces a higher system-level quality score when either sensor retains relatively high perception quality. Here, $Q_{fused}$ is treated as a monotonic surrogate for system availability rather than a calibrated probability of perception success. The fusion gain depends on the spatial correlation between the two quality fields. As established in Section 2.2, $Q_{cam}$ and $Q_{lidar}$ have identical spatial ordering under the pure-smoke model. Therefore, the Camera-Only, Lidar-Only, and QC-A* planners tend to produce qualitatively similar routes, although their exact path lengths and path-quality metrics may differ because their cost magnitudes are not identical. Under these conditions, the fusion benefit is reflected mainly in the pointwise increase in the system-level quality score rather than in a fundamental change in route geometry.

### 3.4. Algorithm Flow

**Figure 3:**
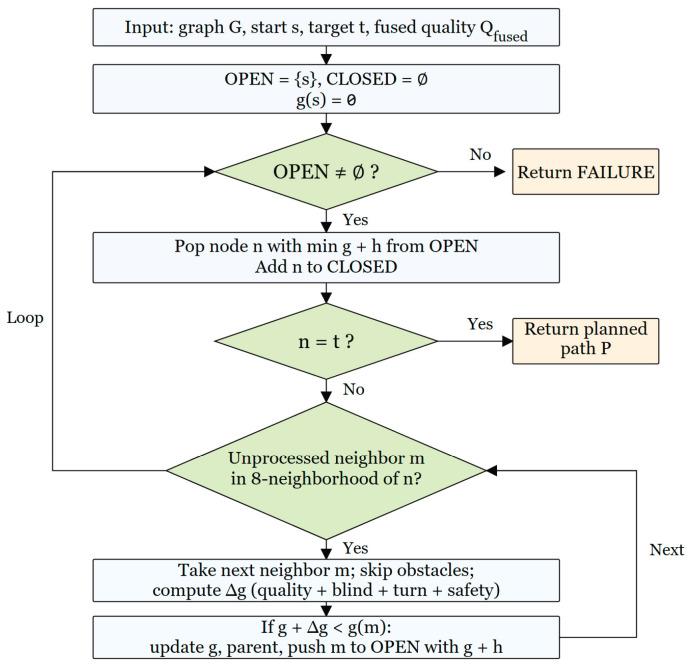
QC-A* path planning algorithm flowchart.

As illustrated in Figure 3, QC-A* follows the basic node-expansion framework of traditional grid-based A*, but modifies the step cost by incorporating perception quality, turning smoothness, and obstacle-clearance considerations. The fused quality field $Q_{fused}$ is stored as an additional grid attribute and is queried during node expansion, so that each candidate node is evaluated according to both geometric distance and local sensing reliability. Euclidean distance is used as the heuristic to guide the search toward the goal. Because the cost function includes a direction-dependent turn-smoothing term and additional safety-related penalties, the method is used as a practical perception-aware planner rather than as a strictly optimal classical A* formulation. All parameters were fixed across the experiments without scenario-specific tuning.

## 4. Experimental Validation and Discussion

### 4.1. Experimental Setup

The environmental parameters are the same as those described in Section 2.1: a 20  m×6  m corridor with a grid resolution of 0.1 m and 21 obstacles. Propane is used as the fuel, and the FDS visibility field is sampled at a height of z=0.3  m. For the straight-corridor experiments, fifty start–goal pairs were randomly generated per scenario using a fixed random seed (42). Start and goal coordinates were uniformly sampled from the traversable area with a minimum Euclidean distance constraint of 8 m, and only pairs successfully planned by all compared methods were retained. The simulations and path-planning experiments are conducted on a computer equipped with an Intel i5 processor (2.20 GHz) and 16 GB RAM using Python 3.10.

**Figure 4:**
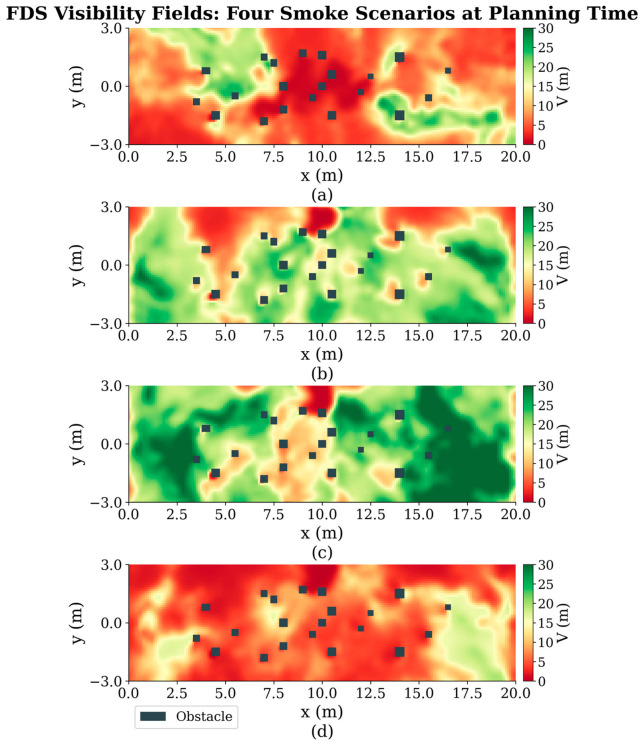
Visibility fields V(x,y) and obstacle layouts for the four FDS scenarios at their respective planning instants. (**a**) Symmetric, t = 24 s; (**b**) Asymmetric, t = 55 s; (**c**) Light-Smoke, t = 43 s; (**d**) Fast-Spread, t = 45 s.

Four physically differentiated scenarios are constructed using FDS 6.9.1 [24], as summarized in Table 2, and their visibility fields are shown in Figure 4. These scenarios are designed to represent different smoke-distribution patterns and severity levels, thereby evaluating whether QC-A* can adapt its planning behavior under both regular and highly constrained smoke conditions. In the Symmetric scenario, 12 burners are arranged symmetrically along the corridor centerline, producing dense smoke near the center and relatively clear regions toward both sides. This scenario serves as the baseline for evaluating planner behavior under a regular smoke distribution.

The Asymmetric scenario uses an offset fire source, producing substantially lower visibility in the upper part of the corridor than in the lower part. It is used to evaluate the planner’s ability to identify and exploit clearer lateral regions when the smoke field is spatially biased.

Although the Light-Smoke scenario has a relatively high HRRPUA (Heat Release Rate Per Unit Area), its spatially localized smoke distribution, lower soot yield, and selected snapshot time result in approximately 85% of the field having visibility above 10 m. This scenario tests whether QC-A* remains close to the geometrically shortest path without producing unnecessary detours under relatively clear conditions.

The Fast-Spread scenario uses the highest HRRPUA and the highest soot yield. At the selected snapshot, approximately 43% of the field has visibility below 5 m, leaving only narrow low-smoke corridors. This scenario evaluates the planner’s ability to exploit limited clearer regions under rapidly developing and extremely dense smoke.

Before path planning, the visibility field of each scenario is converted into the corresponding camera, Lidar, and fused sensor-quality fields using the physical model described in Section 2.2. These quality fields provide the perception-aware inputs for the subsequent path-planning comparison.

To provide a hierarchical comparison, five methods with different cost formulations are evaluated, as summarized in Table 3. The five methods are selected to form a structured ablation study: (1) Traditional A* [11] serves as the baseline for purely geometric planning; (2) Smoke-Weighted A* represents the conventional heuristic approach of using raw visibility maps for cost evaluation, typical in fire-evacuation routing; (3) Camera-Only and (4) Lidar-Only QC-A* serve as single-sensor baselines to demonstrate the limitations of individual modalities; and (5) Fused QC-A* represents the proposed multi-sensor approach. This structure verifies that the proposed method’s benefits arise from the physical modeling and fusion rather than the underlying search algorithm alone. Camera-Only, Lidar-Only, and QC-A* use the same turning, obstacle-clearance, and blind-zone penalties, differing only in the perception-quality field used in the cost function.

Traditional A* serves as the geometric reference without considering perception safety. Smoke-Weighted modifies the edge cost directly using visibility V, defined as d[1+βK/V], where β=0.5. However, it does not explicitly model sensor-specific degradation or blind-zone thresholds. It also cannot represent wavelength-dependent extinction or exploit the complementary characteristics of heterogeneous sensors and does not include turning or obstacle-clearance penalties. Camera-Only and Lidar-Only replace $Q_{fused}$ with $Q_{cam}$ and $Q_{lidar}$, respectively, while retaining the other cost terms of QC-A*. They are used to evaluate the contribution of multi-sensor fusion relative to single-sensor planning. QC-A* uses $Q_{fused}$ to construct the complete perception-aware cost. All five methods use the same A* search framework. In the sensor-failure stress tests described in Section 4.5, the Quality Cost methods are additionally subject to a common cumulative perception-exposure limit of $E_{max}=100.0$.

Fifty start–goal pairs were randomly generated for each scenario using a fixed random seed (42). Start and goal coordinates were uniformly sampled from the traversable area with a minimum Euclidean distance constraint of 8 m, and only pairs successfully planned by all five methods were retained. The evaluation metrics are selected to jointly measure path efficiency and perceptual safety. The evaluation metrics are listed in Table 4. For all methods, $\bar{Q}$ and $Q_{min}$ are calculated using the same $Q_{fused}$ field, representing the perception-safety level that would be obtained if each path were executed by the dual-sensor system. This provides a consistent basis for cross-method comparison.

### 4.2. Parameter Sensitivity Analysis

**Figure 5:**
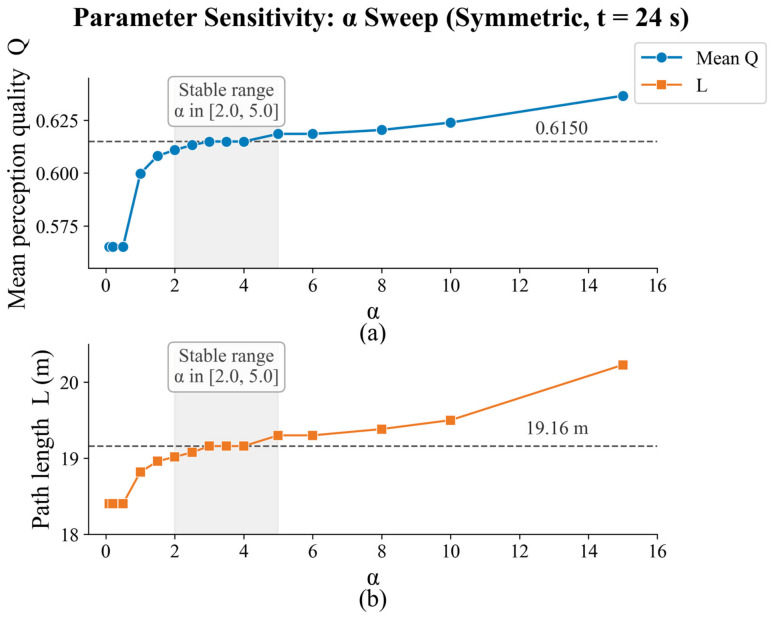
Sensitivity analysis of α in the Symmetric scenario at the 24 s snapshot: (**a**) mean perception quality $\bar{Q}$; (**b**) path length L. The gray region indicates α∈[2,5].

The parameter α primarily controls the trade-off between path length and perception quality. The remaining parameters are fixed according to the adopted safety thresholds, robot-clearance requirements, and exposure constraints. To examine sensitivity to α, its value was varied from 0.1 to 15.0 for a representative start–goal pair, (2.0,−1.0)→(18.0, 1.0), in the Symmetric scenario at the 24 s snapshot. The mean perception quality $\bar{Q}$ and path length L were recorded, as shown in Figure 5.

Within α∈[2.5,4.0], both $\bar{Q}$ and L remain nearly unchanged, with $\bar{Q}=0.613-0.615\;$ and L=19.16 m. Therefore, α=3.0, located within this stable interval, is adopted for all experiments. Because this analysis uses one representative start–goal pair, the identified interval indicates local robustness rather than universal parameter insensitivity.

The remaining parameters are held constant across all experiments: $\beta_{blind}=50.0,\;{\;\beta }_{turn}=0.02,\;{\;d}_{safe}=0.3\;m$, $Q_{blind}=0.25$, and $E_{max}=100.0$. These values respectively control blind-zone avoidance, path smoothness, obstacle clearance, the perception-failure threshold, and cumulative exposure. No scenario-specific parameter adjustment is performed.

### 4.3. Path Visualization Analysis

**Figure 6:**
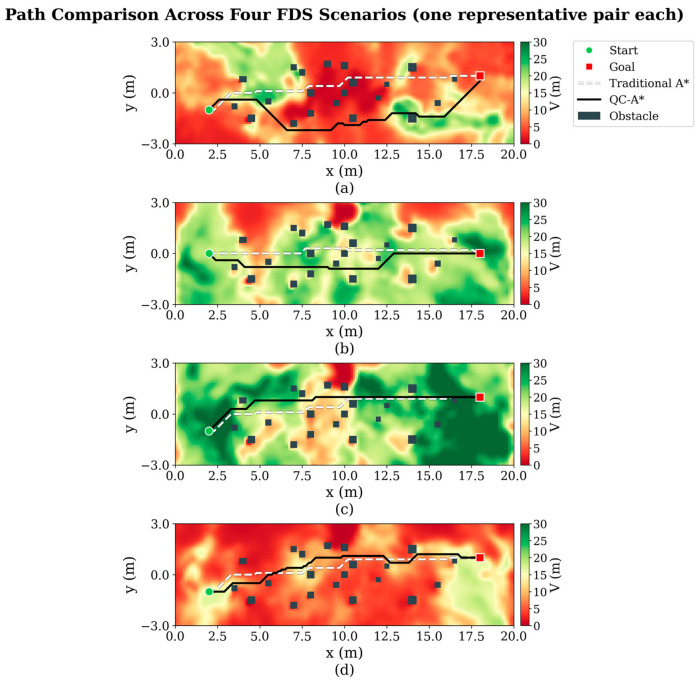
Representative path comparisons in the four scenarios. Traditional A* paths are shown as white dashed lines, and QC-A* paths are shown as black solid lines over the corresponding visibility fields. (**a**) Symmetric scenario. (**b**) Asymmetric scenario. (**c**) Light-Smoke scenario. (**d**) Fast-Spread scenario.

Before presenting the statistical comparison, representative path cases are used to illustrate the behavior of QC-A*. Figure 6 compares Traditional A* and QC-A* for one representative start–goal pair in each of the four scenarios. The results demonstrate how QC-A* responds to spatial variations in perception quality.

In the Symmetric scenario, QC-A* makes a moderate lateral detour around the central low-visibility region while remaining close to the Traditional A* path in clearer areas. In the Asymmetric scenario, QC-A* shifts toward the clearer lower part of the corridor and reduces its traversal through the high-attenuation region. In the Light-Smoke scenario, where perception quality is relatively high over most of the environment, the two paths are nearly coincident, indicating that QC-A* introduces only a negligible detour under relatively clear conditions. In the Fast-Spread scenario, 43% of the field has visibility below 5 m, and only narrow, relatively clear corridors remain. Consequently, the available space for path adjustment is limited, but QC-A* still favors locally higher-quality regions where possible.

Overall, the representative cases show that QC-A* produces more evident path deviations when low-quality regions intersect the geometrically shortest route, while remaining close to Traditional A* in relatively clear regions. The extent of the deviation is constrained by both the spatial distribution of perception quality and the available obstacle-free space. Section 4.4 further evaluates these observations using randomly generated start–goal pairs.

### 4.4. Comprehensive Statistical Analysis

This section quantitatively evaluates the qualitative path behaviors discussed above. Table 5 summarizes the results of the five methods for randomly generated start–goal pairs across the four scenarios.

Table 5 Comparison of key path-quality indicators across the four scenarios. shows a consistent overall trend across the four scenarios. Traditional A* generally produces the shortest paths but the lowest perception-quality metrics. Smoke-Weighted improves perception quality with only a small increase in path length. However, because it directly uses visibility V, it cannot distinguish wavelength-dependent sensor degradation or explicitly represent sensor-specific failure thresholds.

Under the natural-smoke conditions, Camera-Only, Lidar-Only, and QC-A* produce qualitatively similar results because $Q_{cam}$ and $Q_{lidar}$ have identical spatial ordering, as discussed in Section 3.3. QC-A* generally achieves perception-quality metrics comparable to those of the single-sensor methods, although it does not outperform them in every metric. In particular, Camera-Only achieves the highest $\bar{Q}$, $Q_{min}$, and lowest low-visibility exposure ratio in the Asymmetric scenario, whereas QC-A* provides marginally higher $Q_{min}$ in the Symmetric, Light-Smoke, and Fast-Spread scenarios.

In the Symmetric and Asymmetric scenarios, the availability of clearer lateral regions allows the quality-aware methods to reduce traversal through high-attenuation areas. In the Fast-Spread scenario, widespread dense smoke and limited clear space restrict the possible path adjustments, so the improvements mainly arise from local path refinement. In the Light-Smoke scenario, QC-A* remains close to Traditional A*, with a path-length increase of only 0.18 m, indicating limited additional detour under relatively clear conditions.

Across the Symmetric and Asymmetric scenarios, weighted by the number of valid start–goal pairs, QC-A* increases the mean path length by 15.3% while reducing the low-visibility exposure ratio from 40.7% to 19.6%, corresponding to a 51.9% reduction. Meanwhile, the worst-case path perception quality $Q_{min}$ increases from 0.067 to 0.177.

The statistical comparison demonstrates that QC-A* improves perception safety over Traditional A* and Smoke-Weighted across all scenarios while maintaining acceptable path efficiency. In the Symmetric scenario, QC-A* achieves higher $Q_{min}$ values compared with all single-sensor baselines, including Camera-Only and Lidar-Only. In the Asymmetric scenario, Camera-Only achieves the highest individual-sensor metrics, whereas QC-A* provides robust multi-sensor fusion performance without relying on a single sensor modality. Overall, the perception-aware cost successfully guides the planner away from high-attenuation regions. The moderate increase in path length (15.3%) represents a reasonable trade-off for substantially improved perceptual safety. In the Light-Smoke scenario, QC-A* remains close to Traditional A* with negligible additional path length, demonstrating that the method does not overreact to mild smoke conditions. The stress-test results further confirm that multi-sensor fusion provides robustness under sensor-specific failure conditions, with QC-A* maintaining high planning success rates while single-sensor methods degrade significantly.

**Figure 7:**
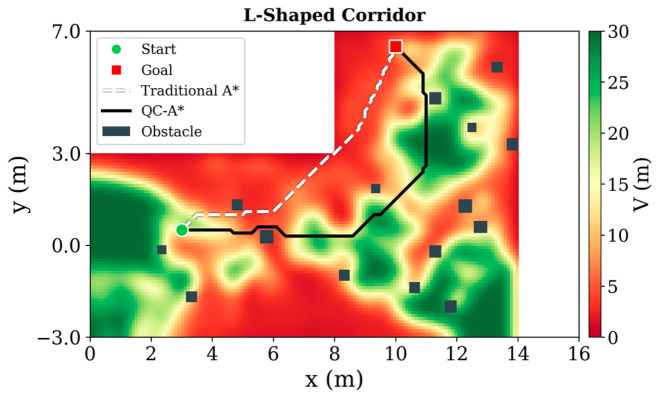
L-shaped corridor generalization test environment and QC-A* planning result.

To further examine whether the proposed quality-cost mechanism is limited to the original straight-corridor layout, an additional L-shaped corridor environment was constructed, as shown in Figure 7. The L-shaped map changes the navigable geometry and obstacle distribution while keeping the grid resolution, sensor-quality modeling procedure, and all cost-function parameters identical to those used in the straight-corridor experiments. Therefore, this test evaluates whether QC-A* can retain its perception-aware planning behavior under a different spatial layout without scenario-specific parameter adjustment. For the L-shaped corridor experiment, thirty start–goal pairs were randomly generated using a fixed random seed (42). Start coordinates were uniformly sampled from x ∈ [1, 5], y ∈ [−2, 2], and goal coordinates from x ∈ [9, 13], y ∈ [3.5, 6.5], with only pairs successfully planned by all compared methods retained.

Across all 30 start–goal pairs, QC-A* simultaneously outperformed both single-sensor methods on all 30 pairs. Compared with Traditional A* which achieved a mean path length of 10.54 m and a mean perception quality $\bar{Q}\;$ of 0.399 with V<5 m of 45.4%, QC-A* improved $\bar{Q}$ to 0.803 and reduced V<5 m to 4.0%, at a moderate path length of 12.97 m. Camera-Only and Lidar-Only achieved $\bar{Q}\;$ values of 0.561 and 0.645 with path lengths of 14.04 m and 13.49 m, both lower than QC-A* in perception quality despite comparable or greater path length costs. The L-shaped geometry constrains all feasible paths to pass through the corridor junction, which amplifies the penalty incurred by any single-sensor strategy that cannot exploit the complementary clean regions on both sides of the junction. QC-A* leverages this complementarity by routing through the side offering higher fused quality at each point along the path. Representative paths are visualized in Figure 7, where Traditional A* traverses the shortest geometric route directly through high-attenuation areas, whereas QC-A* deviates toward regions of higher fused quality while maintaining a comparable path length. These results, obtained without any parameter re-tuning, confirm that the fixed parameter set generalizes from a straight to an L-shaped corridor geometry and that the quality-cost mechanism consistently produces perception-aware paths across different environment layouts.

### 4.5. Constructed Sensor Failure Stress Test

Under pure-smoke conditions, QC-A* and the single-sensor methods tend to generate similar paths because their quality fields share the same spatial ordering, as discussed in Section 4.4. To evaluate fusion under sensor-specific degradation, idealized failure regions are superimposed on the quality fields of the four FDS scenarios: flame-glare regions, where $Q_{cam}$ is set to 0.01 while $Q_{lidar}$ remains unchanged, and water-mist regions, where $Q_{lidar}$ is set to 0.01 while $Q_{cam}$ remains unchanged. Two glare regions and two water-mist regions, each measuring 6  m×2  m, are placed in each scenario, covering 21–43% of the map area. Because FDS does not directly model these sensor-specific effects, the constructed regions serve as controlled stress tests rather than direct physical simulations.

**Figure 8:**
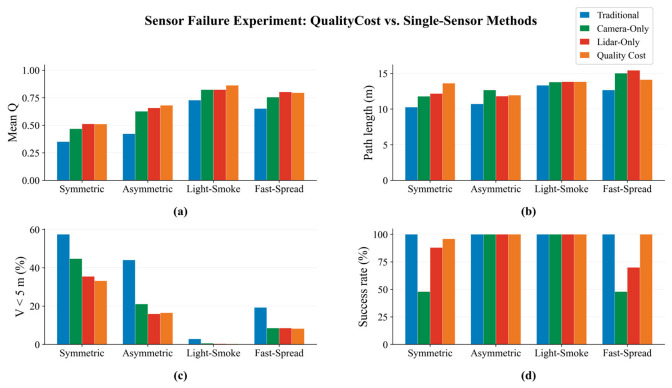
Results of the constructed sensor-failure stress tests across the four scenarios: (**a**) mean system-availability score $\bar{Q}$; (**b**) path length L; (**c**) low-visibility exposure ratio V<5 m (%); (**d**) planning success rate.

A cumulative perception-exposure budget is used as the path-executability criterion by summing $1-Q_{m}$ over all nodes along a path, where $Q_{m}$ denotes $Q_{cam}$, $Q_{lidar}$, or $Q_{fused},$ depending on the method. A search branch is pruned when its cumulative exposure exceeds $E_{max}=100.0,$ which corresponds approximately to 100 fully degraded grid nodes or 10 m of traversal at the 0.1 m grid resolution. If no feasible branch remains, the trial is recorded as unsuccessful. Fifty random start–goal pairs with a fixed seed of 42 are evaluated for each scenario, and the results are shown in Figure 8.

Figure 8 shows that QC-A* benefits from the complementary availability of the two sensors. In a camera-specific failure region, Lidar can retain useful quality, whereas the camera can remain available in a Lidar-specific failure region. QC-A* therefore generally maintains a higher fused-quality score than either single-sensor method when their respective failure regions intersect a path.

Additional pairwise analysis shows that QC-A* simultaneously outperforms both single-sensor methods in $\bar{Q}$ for 59% of the evaluated Light-Smoke pairs and 24% of the evaluated heavy-smoke pairs. Relative to Traditional A*, its path length increases by 32.6% in the Symmetric scenario but by less than 1 m in the other scenarios. The low-visibility exposure ratio is reduced from the 19–57% range of the compared methods to 8–33% in the heavy-smoke cases. Camera-Only achieves only a 48% planning success rate in the Symmetric scenario, while Lidar-Only decreases to 70% in Fast-Spread. In comparison, QC-A* maintains a success rate of 96–100% across all four scenarios. These results indicate that fusion provides limited additional benefit when both sensors remain available but substantially improves robustness under sensor-specific failure conditions.

## 5. Conclusions

QC-A* was proposed as a perception-aware path-planning method for mobile robots operating in smoke-filled environments. The main conclusions are as follows:

(1) The results demonstrate that physically derived perception-quality fields can effectively connect smoke-induced sensor degradation with navigation decisions, providing a more interpretable basis for path planning than purely empirical environmental penalties.
(2) QC-A* adapts its behavior to different smoke distributions. In the Light-Smoke scenario, its path length is 13.51 m, only 0.18 m longer than that of Traditional A*, indicating a limited additional detour under relatively clear conditions.
(3) The improvement is more evident when clearer alternative regions are available. In the Symmetric scenario, QC-A* increases $Q_{min}$ from 0.024 to 0.150 and reduces low-visibility exposure from 43.4% to 23.7%. In the Asymmetric scenario, the corresponding exposure decreases from 38.7% to 16.5%.
(4) Multi-sensor fusion contributes most when the camera and Lidar exhibit spatially distinct failure modes. Additional pairwise analysis shows that QC-A* outperforms both single-sensor methods in perception quality for 59% of the Light-Smoke pairs and 24% of the heavy-smoke pairs. Its additional path-level benefit is comparatively limited when the two sensors undergo correlated pure-smoke degradation.

The present study was conducted under a two-dimensional environment, accurate robot localization, and idealized sensor-specific failure regions. Future work will validate the model through controlled fire experiments, extend it to three-dimensional smoke environments, and investigate adaptive online replanning under dynamically evolving conditions.

## Figures and Tables

**Table 1:** Core parameters of the physical modeling chain.

Parameter	Symbol	Value	Unit
Koschmieder constant	K	3.0	—
Visible mass extinction coefficient	$\sigma_{vis}$	8.7	$m^{2}/g$
Near-infrared mass extinction coefficient	$\sigma_{nir}$	6.1	$m^{2}/g$
Reference perception distance	$d_{ref}$	3.5	m
Perception blind-zone threshold	$Q_{blind}$	0.25	—

**Table 2:** FDS fire-source parameters for the four scenarios.

Scenario	Fire-Source Configuration	Location	HRRPUA ($kW/m^{2}$)	Soot Yield	Planning Time (s)	Smoke Characteristics
**Symmetric**	12 burners arranged along the centerline	x∈[9.0,11.0],y=0	500	0.08	24	Symmetric distribution with dense central smoke and clearer lateral regions
**Asymmetric**	Offset fire source	x∈[9.5,10.5], y∈[2.0,3.0]	1500	0.15	55	Denser smoke in the upper region and clearer conditions in the lower region
**Light-Smoke**	Localized fire source in the upper half	x∈[9.0,11.0],y>0	2000	0.08	43	Localized light smoke used to evaluate unnecessary detours
**Fast-Spread**	Offset fire source	x∈[9.5,10.5], y∈[2.0,3.0]	3000	0.25	45	Rapidly developing dense smoke with narrow low-smoke corridors

**Table 3:** Edge-cost formulations of the compared methods.

Method	Perception-Related Cost Term	Core Parameters	Physical Meaning
Traditional A*	d(c,n)	—	Pure geometric-distance cost
Smoke-Weighted	d(c,n)[1+βK/V(n)]	β=0.5 m,K=3.0	Empirical inverse-visibility penalty
Camera-Only	$d(c,n)[1+\alpha (1-Q_{cam}(n))]$	α=3.0	Camera-based perception-quality cost
Lidar-Only	$d(c,n)[1+\alpha (1-Q_{Lidar}(n))]$	α=3.0	Lidar-based perception-quality cost
Quality Cost A*(QC-A*)	$d(c,n)[1+\alpha (1-Q_{fused}(n))]$	α=3.0	Multi-sensor fused perception-quality cost

**Table 4:** Path quality evaluation metrics.

Metric	Symbol	Definition	Meaning
Mean system availability score	$\bar{Q}$	Mean of $Q_{fused}$ over path nodes	Perceptual safety level
Worst-case system availability score	$Q_{min}$	Cross-pair mean of the minimum $Q_{fused}$ along each path	Safety lower bound
Path length	L	Total path length (m)	Detour cost
Low-visibility hazard zone proportion	V<5 m	Percentage of nodes with visibility below 5 m	Hazard zone exposure

**Table 5:** Comparison of key path-quality indicators across the four scenarios.

Scenario	Method	L (m)	$\bar{Q}$	$Q_{min}$	V < 5 m (%)
Symmetric (N=32)	Traditional	10.62	0.435	0.024	43.4
	Smoke-Weighted	10.67	0.510	0.066	37.6
	Camera-Only	12.48	0.583	0.122	23.6
	Lidar-Only	13.25	0.587	0.149	22.4
	Quality Cost	13.29	0.585	0.150	23.7
Asymmetric (N=43)	Traditional	11.06	0.486	0.100	38.7
	Smoke-Weighted	11.21	0.644	0.180	21.2
	Camera-Only	12.01	0.706	0.209	13.4
	Lidar-Only	12.02	0.699	0.196	14.2
	Quality Cost	11.98	0.693	0.197	16.5
Light-Smoke (N=49)	Traditional	13.33	0.853	0.627	1.0
	Smoke-Weighted	13.33	0.873	0.699	0.5
	Camera-Only	13.48	0.879	0.718	0.1
	Lidar-Only	13.48	0.879	0.716	0.1
	Quality Cost	13.51	0.880	0.728	0.1
Fast-Spread (N=50)	Traditional	12.68	0.743	0.021	18.4
	Smoke-Weighted	12.70	0.770	0.025	16.0
	Camera-Only	13.78	0.837	0.050	8.4
	Lidar-Only	13.78	0.836	0.050	8.4
	Quality Cost	13.75	0.836	0.052	8.4

Note: N denotes the number of valid start–goal pairs. Metrics are defined in Table 4.

## Data Availability

The data presented in this study are available on request from the corresponding author.
